# Effect of first-line biologic initiation on glucocorticoid exposure in children hospitalized with new-onset systemic juvenile idiopathic arthritis: emulation of a pragmatic trial using observational data

**DOI:** 10.1186/s12969-021-00597-z

**Published:** 2021-07-05

**Authors:** Rosemary G. Peterson, Rui Xiao, Hannah Katcoff, Brian T. Fisher, Pamela F. Weiss

**Affiliations:** 1 Children’s Hospital of Philadelphia, Division of Rheumatology, Philadelphia, PA USA; 2 Center for Pediatric Clinical Effectiveness, Children’s Hospital of Philadelphia Research Institute, Philadelphia, PA USA; 3 Dell Children’s Medical Center, Strictly Pediatrics Building, 1301 Barbara Jordan Blvd, Suite 400, Austin, TX 78723 USA; 4 Center for Clinical Epidemiology and Biostatistics, Perelman School of Medicine at the University of Pennsylvania, Philadelphia, PA USA; 5 Children’s Hospital of Philadelphia, Division of Infectious Diseases, Philadelphia, PA USA; 6 Perelman School of Medicine at the University of Pennsylvania, Philadelphia, PA USA

**Keywords:** Juvenile systemic arthritis, Biological therapy, Glucocorticoids

## Abstract

**Background:**

Glucocorticoid exposure is a significant driver of morbidity in children with systemic juvenile idiopathic arthritis (sJIA). We determined the effect of early initiation of biologic therapy (IL-1 or IL-6 inhibition) on glucocorticoid exposure in hospitalized patients with new-onset sJIA.

**Methods:**

We emulated a pragmatic sequence of trials (“pseudo-trials”) of biologic initiation in children (≤ 18 years) hospitalized with new-onset sJIA utilizing retrospective data from an administrative database from 52 tertiary care children’s hospitals from 2008 to 2019. Eligibility window, treatment assignment and start of follow-up between biologic and non-biologic study arms were aligned for each pseudo-trial. Patients in the source population could meet eligibility criteria at several timepoints. Mixed-effects logistic regression was used to determine the effect of biologic initiation on in-hospital glucocorticoid exposure.

**Results:**

Four hundred sixty-eight children met eligibility criteria, of which 19% received biologic therapy without preceding or concomitant initiation of immunomodulatory medications. This proportion significantly increased over time during the study period (*p* <  0.01). 1451 trial subjects were included across 4 pseudo-trials with 71 assigned to the biologic arm and 1380 assigned to the non-biologic arm. After adjustment, there was a trend toward decreased odds of glucocorticoid initiation in the biologic arm compared to the non-biologic arm (OR 0.39, 95% CI [0.13, 1.15]).

**Conclusion:**

Biologic initiation in children hospitalized with new-onset sJIA significantly increased over time and may be associated with reduced glucocorticoid exposure. The increasing use of first-line biologic therapy may lead to clinically relevant reductions in treatment-related adverse effects of glucocorticoid-reliant therapeutic approaches.

## Background

Systemic juvenile idiopathic arthritis (SJIA) is widely considered the most severe subtype of JIA. It is best characterized as an autoinflammatory condition with prominent systemic features of daily high-spiking fevers, evanescent rashes, hepatosplenomegaly, lymphadenopathy, and serositis. Until recently, glucocorticoid-reliant treatment approaches were necessary due to ineffectiveness of biologic and synthetic disease-modifying antirheumatic drugs (DMARDs) used for other subtypes of JIA, including TNF-inhibitors and methotrexate [1–3].

Although effective in controlling the acute inflammation of sJIA, glucocorticoid use in children often results in short and long-term adverse effects, including growth inhibition, osteoporosis, avascular necrosis, obesity, cataracts, hypertension and psychologic pathology [4, 5]. In 2012, randomized controlled trials (RCTs) demonstrated that IL-1 and IL-6 inhibitors were efficacious in controlling the systemic and articular manifestations of sJIA [6–8]. Based on these findings, the 2013 American College of Rheumatology (ACR) recommendations for sJIA treatment endorsed IL-1 inhibition as initial therapy for patients with severe disease [9]. This recommendation represented a major turning point in the treatment approach to sJIA.

There is now mounting evidence that early initiation of biologic therapy (specifically IL-1 or IL-6 inhibition) not only improves disease course but also has the additive effect of reducing glucocorticoid exposure [10–14]. In single-center observational studies, up to 90% of children treated with first-line IL-1 inhibition monotherapy did not require adjunctive glucocorticoid therapy after at least 1 year of follow up [10, 11, 14]. However, it is not known whether early initiation of biologic therapy will reduce the need for glucocorticoids among children hospitalized at the onset of sJIA. These children often have more severe disease and thus may still need glucocorticoids to control their inflammation in this index admission. Prior work by our group found significant variation across hospitals in the use of biologic and glucocorticoid therapy for children presenting with sJIA between 2008 and 2019 [15]. This variation in practice represents an opportunity to assess whether the use of a biologic as first line therapy is causally associated with a reduction in glucocorticoid use.

The primary objective of this study was to determine whether early initiation of first-line biologic therapy in hospitalized patients with new-onset sJIA patients results in a reduction in glucocorticoid exposure during the same hospitalization. SJIA is a rare disease, limiting the feasibility of conducting an RCT and necessitating the use of observational data. We utilized a novel strategy that leverages observational data to emulate a hypothetical pragmatic trial (pseudo-trial). In this approach treatment assignment, specification of eligibility, and start of follow up between biologic initiator and non-initiator study arms are aligned [16, 17]. In addition to emulating a pragmatic trial, this methodology aims to reduce the risk of immortal time bias and selection bias that commonly arise in observational studies.

## Methods

This was an emulated pragmatic trial using observational data of children hospitalized with new-onset sJIA. This study was reviewed and determined to be exempt by the Children’s Hospital of Philadelphia (CHOP) Internal Review Board.

### Data source

The data source was diagnostic and billing records from the Pediatric Health Information System (PHIS) captured between 1/1/2008–3/31/2019. PHIS is an administrative database that contains inpatient, emergency department, ambulatory surgery, and observation unit information from 52 not-for-profit, tertiary care pediatric hospitals. Data include demographics, dates of service, discharge disposition, and daily inpatient billing data for medications. Records are linked longitudinally across admissions via unique patient identifiers. Data are deidentified at the time of submission and data quality is assured through a joint effort between the Children’s Hospital Association and participating hospitals.

### Outcomes

The primary study outcome was initiation of glucocorticoids during the index hospitalization for sJIA. Glucocorticoid exposure was defined as having an inpatient billing code for at least one of the following: oral or intravenous dexamethasone, hydrocortisone, methylprednisolone, prednisolone, or prednisone. This outcome was assessed during a period that started on day 2 of each pseudo-trial (time zero) and ended at index hospitalization discharge. Secondary outcomes included exposure to pulse dose glucocorticoids (~ 30 mg/kg/dose, maximum 1 g) and billing for a glucocorticoid on the last full hospital day (as a proxy for discharge on glucocorticoids).

### Medications

Medication usage was defined using pharmacy billing data. Biologic therapy included anakinra, rilonacept, canakinumab, or tocilizumab. Scheduled non-steroidal anti-inflammatory drug (NSAID) use was defined as a code for ibuprofen, naproxen, indomethacin, piroxicam, diclofenac, meloxicam, or celecoxib on two or more consecutive hospital days. Oral and subcutaneous methotrexate and intravenous and subcutaneous tocilizumab were pooled as methotrexate and tocilizumab, respectively.

### Study population and pseudo-trial design

**Fig. 1:**
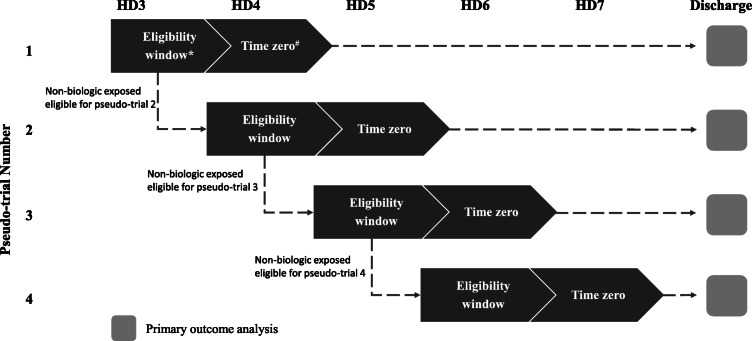
Flowchart of patient enrollment into pseudo-trials. HD = hospital day, * Treatment assigned during eligibility window. # Time zero represents the start of follow up

The source population consisted of 534 children (≤ 18 years) discharged from one of 52 PHIS hospitals between 1/1/2008 and 3/31/2019 with new-onset sJIA. The algorithm to identify children with new-onset sJIA from the PHIS database was previously validated [15]. Although the International League of Associations for Rheumatology (ILAR) criteria for systemic JIA officially only includes patients under the age of 16, the age range for this study was extended to age 18 to maximize study population given that adult onset still’s disease (AOSD) is thought to be clinically equivalent to sJIA [18]. To avoid inclusion of sJIA patients admitted for a disease flare, the cohort definition excluded patients who received glucocorticoids, biologics, or methotrexate in the first 2 hospital days. We emulated a pragmatic sequence of trials or pseudo-trials by aligning the eligibility window, treatment assignment and start of follow-up between biologic and non-biologic study arms [19, 20]*.* (Fig. 1) All patients were subject to the same eligibility criteria of no glucocorticoid exposure prior to the start of follow-up. For each trial, patients who initiated a biologic medication during the one-day baseline evaluation window were considered biologic initiators (exposed). Those who did not receive biologic therapy were considered biologic non-initiators (unexposed). Exposure analysis paralleled an intention-to-treat design. Patients in the source population could meet eligibility criteria at several timepoints and could contribute to up to 4 pseudo-trials; however, once a biologic was initiated, the patient was no longer eligible for subsequent trials. This method increases statistical efficiency when subjects fulfill eligibility criteria at multiple timepoints and can therefore be considered exposed and unexposed at different times [19–22].

### Covariates

Baseline covariates included demographics (age, sex, race, commercial insurance), hospital characteristics (region, total annual patient volume), year of hospital discharge and disease severity indicators during the first two hospital days. These disease severity indicators included intensive care unit (ICU) stay, supplemental oxygen, laboratory billing code for blood gas, and laboratory billing code for multiple complete blood counts in a single hospital day. Macrophage activation syndrome (MAS) was not included in the model as MAS is only indicated as a discharge diagnosis and may have occurred before or after the timing of the primary outcome of glucocorticoid initiation.

### Statistical analysis

Standard descriptive statistics including range, mean and standard deviation (SD) for normally distributed variables or median and interquartile range (IQR) for non-normally distributed variables were used, as appropriate. Kruskal-Wallis and chi-squared tests were used to assess for baseline demographic and clinical differences between unique participants in study arms. Trends in treatment over time were assessed using an extension of the Wilcoxon rank-sum test for trends.

In an effort to mimic randomization and minimize confounding by indication, we first estimated the propensity to receive biologics (p-score) using a logistic regression model conditional on the baseline covariates described above. The resulting p-score ranged from 0 to 100, with higher p-score indicating a greater propensity to receive biologic. For the pseudo-trial analyses to assess the association of biologic exposure with the primary and secondary outcomes, we used mixed-effects logistic regression models. The mixed-effects logistic regression model was inclusive of the p-scores as well as baseline covariates that were unbalanced between the two arms within p-score tertiles. A hospital-specific and subject-specific random effect were included to account for within-hospital clustering and the possibility that patients may contribute to multiple pseudo-trials, respectively. The biologic exposure effect on the primary outcome was presented as an odds ratio (OR) with 95% confidence interval (CI). Sensitivity analyses were done by excluding patients who did not receive scheduled NSAIDs during the eligibility window of each pseudo-trial to emulate common clinical practice of first-line NSAID treatment prior to considering escalation in therapy.

All analyses were performed using SAS 9.4 (Cary, NC: SAS Institute Inc) and Stata 15 (StataCorp. 2017, *Stata Statistical Software.* College Station, TX: StataCorp LP).

## Results

During the study period, 66 children received glucocorticoid therapy prior to the start of follow up, leaving 468 children who met eligibility criteria and were discharged from one of 52 PHIS contributing institutions with new-onset sJIA. Overall, 19% of patients had a billing code for biologic therapy without preceding or concomitant initiation of adjunctive immunomodulatory medications (i.e. methotrexate, glucocorticoids). This proportion increased significantly over the study period from 2008 to 2019 (*p* <  0.01), reaching 40% of patients discharged between 2017 and 2019. The median time to start a biologic medication from admission was 4 days (IQR 3 to 7). 51.3% of patients received glucocorticoids, including 22.5% that received at least one dose of pulse dose glucocorticoids. The median time from admission to start of glucocorticoids was 5 days (IQR 3 to 7). Of those who were started on glucocorticoids, 92.1% continued to receive them until hospital discharge.

Table 1 displays comparisons of baseline demographic and clinical characteristics between biologic initiator and non-initiator patients. Compared to the biologic non-initiators, biologic initiators were more likely to have multiple complete blood counts (CBCs) drawn within the first or second hospital day (*p* = 0.03). Methotrexate exposure during hospitalization was more common in the biologic non-initiators compared to biologic initiators (8.6% vs. 2.8%, *p* = 0.09) while scheduled NSAID exposure was balanced between groups (87.7% vs. 81.7%, *p* = 0.17).

**Table 1:** Baseline characteristics of initiators and non-initiators of first-line biologic therapy

	Initiators (***n*** = 71)	Non-initiators (***n*** = 397)	***p***-value
**Demographics**			
Age, median (IQR)	7.0 (4.0, 11.0)	6.0 (3.0, 12.0)	0.34
Sex (male), N (%)	41 (57.7%)	203 (51.1%)	0.30
Race, N(%)			0.66
White	43 (60.6%)	265 (66.8%)	
Black	12 (16.9%)	49 (12.3%)	
Asian	3 (4.2%)	12 (3.0%)	
Other	13 (18.3%)	71 (17.9%)	
Commercial insurance, N(%)	163 (41.1%)	33 (46.5%)	0.39
**Hospital Characteristics**			
Hospital region, N(%)			0.83
Northeast	15 (21.1%)	71 (17.9%)	
Southeast	13 (18.3%)	69 (17.4%)	
Southwest	10 (14.1%)	44 (11.1%)	
Midwest	18 (25.4%)	120 (30.2%)	
West	15 (21.1%)	93 (23.4%)	
Hospital volume, N(%)			< 0.01
Low volume (≤ 12,000)	3 (4.2%)	39 (9.8%)	
Medium low volume (12,000-15,999)	5 (7.0%)	88 (22.2%)	
Medium high volume (16,000-21,000)	28 (39.4%)	131 (33.0%)	
High volume (≥21,000)	35 (49.3%)	139 (35.0%)	
**Clinical Features Prior to Treatment Assignment**			
Disease Severity Indicators^a^			
ICU level of care	5 (7.0%)	15 (3.8%)	0.21
Multiple complete blood counts	10 (14.1%)	26 (6.5%)	0.03
Blood gas	6 (8.5%)	17 (4.3%)	0.13
Supplemental oxygen	3 (4.2%)	16 (4.0%)	0.94

Legend. ^a^ Within the first two hospital days of admission

The comparison of glucocorticoid receipt by biologic initiators and biologic non-initiators included 1451 trial “subjects” across the 4 pseudo-trials. Biologic initiator and non-initiator assignment status for each pseudo-trial is shown in Table 2. Of the 71 biologic initiators, 70 received an IL-1 inhibitor (69 received anakinra, one received canakinumab), and one received an IL-6 inhibitor (tocilizumab). Nineteen patients initiated biologic therapy after the 4th pseudo-trial’s baseline evaluation period and were included in the biologic non-initiator arm for all trials due to the intention-to-treat study design. A lower proportion of these patients subsequently received glucocorticoids compared to the other patients in the non-biologic initiator arm (36.8% vs. 56.9%, *p* = 0.09).

**Table 2:** Patient enrollment in individual and pooled pseudo-trial study arms

	Initiators	Non-initiators
Pseudo-trial 1	*N* = 29	*N* = 439
Pseudo-trial 2	*N* = 18	*N* = 360
Pseudo-trial 3	*N* = 12	*N* = 313
Pseudo-trial 4	N = 12	*N* = 268
**Pooled pseudo-trials**	**Total = 71**	**Total = 1380**

Results from unadjusted and adjusted analyses for the primary outcome are displayed in Table 3. The p-score was informed by discharge year, hospital volume, hospital region, commercial insurance, age, gender, and disease severity indicators within first two hospital days (multiple CBCs, ICU level of care, blood gas, supplemental oxygen). Commercial insurance, gender, race, and discharge year were unbalanced within propensity score tertiles. Therefore, the p-score and these covariates were included in the adjusted model. After adjustment, there was a trend toward decreased odds of glucocorticoid initiation in the biologic arm compared to the non-biologic arm, although it did not reach statistical significance (OR 0.39, 95% CI [0.13, 1.15]). Sensitivity analysis excluding patients who did not receive scheduled NSAIDs during the eligibility window yielded similar results with an unadjusted OR of 0.38 (95% CI [0.12, 1.21]) for glucocorticoid exposure in the biologic arm. For secondary outcomes, there was a trend toward decreased likelihood to receive pulse dose glucocorticoids and glucocorticoid on the last hospital day in the biologic arm but it did not reach statistical significance (OR 0.67, 95% CI [0.15, 3.01] and OR 0.44, 95% CI [0.15, 1.31], respectively).

**Table 3:** Unadjusted and propensity-adjusted odds ratios for association of first-line biologic treatment with subsequent glucocorticoids

	Unadjusted OR (95% CI)	Adjusted OR (95% CI)^a^
Glucocorticoid initiation	0.40 (0.15, 1.09)	0.39 (0.12, 1.15)
Glucocorticoids on last hospital day	0.43 (0.16, 1.14)	0.44 (0.15, 1.31)
Pulse dose glucocorticoids	0.86 (0.24, 3.16)	0.67 (0.15, 3.01)

Legend. ^a^ Adjusted model includes hospital site and subject as random effects and propensity score, admission year, sex, race and commercial insurance as fixed effects

## Discussion

This study evaluated the effect of IL-1 or IL-6 inhibitor biologic use on subsequent glucocorticoid initiation in 468 hospitalized children with new-onset sJIA across 52 geographically diverse US children’s hospitals. Our study highlights important findings regarding the effect of biologic treatment in the inpatient setting. First, while not statistically significant, there was a trend toward decreased odds of glucocorticoid exposure among patients that received initial therapy with a biologic. Second, initial therapy with a biologic without concomitant or antecedent immunomodulatory agents became more common over time although, surprisingly, it was still used in a minority of children diagnosed in the inpatient setting.

Using a validated novel strategy, our study emulated a hypothetical pragmatic trial of biologic initiation. A primary advantage of biologic treatment is the potential to avoid glucocorticoid exposure, which historically, has been a significant driver of morbidity in children with sJIA. The primary analysis found that the use of biologics was associated with a reduction in glucocorticoid use. While this reduction was not statistically significant, a point estimate for the OR of 0.39 is suggestive of clinically important reduction. Importantly, this finding is in accordance with other smaller observational studies that suggested a similar reduction in need for glucocorticoids in patients receiving biologics. In 2010, Nigrovic and colleagues published a series of 46 patients treated with anakinra for new-onset sJIA [10]. Ten of these patients were treated with anakinra monotherapy, of which only one patient required adjunctive glucocorticoids. Subsequent prospective observational studies continued to show positive outcomes with biologic monotherapy with between 65 and 75% of patients achieving sustained clinical remission without adjunctive glucocorticoids [11, 14]. Taken collectively, these observational data suggest a causal association between initial therapy with a biologic agent and subsequent lack of need for glucocorticoids.

The frequency of treatment with first-line biologic therapy without preceding or concomitant initiation of other immunomodulatory agents, particularly glucocorticoids and methotrexate, is increasing and now used in up to 40% of hospitalized patients in recent years. These findings are also in agreement with the initial reports of consensus treatment plan (CTP) utilization in the Childhood Arthritis and Rheumatology Research Alliance (CARRA) pilot study, in which 33% of patients were treated with biologic monotherapy at disease onset [23]. The current draft of the ACR 2020 JIA guidelines explicitly recommend biologic DMARDs (IL-1 and IL-6 inhibitors) as first-line monotherapy and avoidance of oral glucocorticoid monotherapy (grade C recommendations) [24]. Paralleling this recent recommendation, we have shown an increasing trend toward biologic monotherapy, which is encouraging as it should reduce the negative consequences of glucocorticoid-reliant therapeutic approaches. That said, first-line biologic monotherapy is still only trialed in a minority of patients hospitalized with new-onset systemic JIA, highlighting the significant work that needs to be done to identify and address barriers to widespread implementation of these treatment recommendations.

There were several limitations to our study. First, it is important to note that this study was unable to evaluate treatment administered after hospital discharge. That said, the goal of the study was to assess the association of biologic initiation on subsequent in-hospital glucocorticoid exposure. In-hospital exposure is likely a reasonable proxy for outpatient glucocorticoid treatment as nearly all patients started on glucocorticoids received them until date of hospital discharge. While we are unable to determine rates of long-term glucocorticoid exposure, even in the short-term, glucocorticoid-related side effects such as weight gain, mood and sleep disturbances can be debilitating for patients. Hospital readmission for infection was considered as one proxy for treatment-related serious adverse effect, but we did not have sufficient power as only 7 patients were readmitted for infection within 90 days of discharge. Second, our power to detect an effect of biologics on decreasing glucocorticoid initiation may have been reduced by the inclusion of 19 patients who started biologics after the eligibility window of the 4th pseudo-trial in the non-biologic initiator arm. Time to biologic initiation in these patients was too widely dispersed to include in the biologic initiator arm while still adhering to the hypothetical pragmatic trial design. By including these patients in the non-biologic exposed arms, we likely biased ourselves toward the null hypothesis. Third, although this study utilized a novel methodology to emulate a pragmatic trial, it is important to note that this is not equivalent to a RCT in that treatment groups are not randomized, and covariates and outcomes are limited to those accessible within an administrative claims database. As such, there was confounding by indication. We attempted to reduce confounding by indication using propensity score methodology that incorporated multiple baseline demographic, hospital and clinical characteristics that may affect exposure assignment. Despite these efforts residual unmeasured confounding is certain to have existed. While it is impossible to know with certainty the direction of unmeasured confounding, we anticipate that patients who initiated biologic therapy would have been sicker. As such we hypothesize that unmeasured confounding would have further biased us towards the null. There is also the potential for misclassification of the cohort, which is an inherent risk in epidemiologic studies utilizing administrative claims databases in which identification of patients is based on diagnostic codes. To address the potential for misclassification, we utilized a previously validated cohort identification process for children hospitalized with new-onset systemic JIA within PHIS [15]. Fourth, diagnosis of MAS was not included as a balancing metric for the two exposure groups as we could not determine from the administrative data if MAS onset was before the decision to initiate a biologic. However, the frequency of a discharge international classification of diseases (ICD) code for MAS was balanced between study arms suggesting that there was not significant imbalance in this measure by exposure group. Fifth, it is possible that some patients were still undergoing diagnostic work up for sJIA at the time of enrollment in the pseudo-trials as the exact timing of diagnosis is unable to be ascertained from PHIS. To address this concern, we performed a sensitivity analysis excluding patients who had not yet started scheduled NSAIDs, which is the first-line therapy for new-onset sJIA. In the sensitivity analysis, a similar point estimate for the primary study outcome was identified. Finally, although there is currently clinical equipoise between IL-1 and IL-6 inhibitors in treatment of sJIA, the biologic arm of this study only included one patient treated with IL-6 inhibition. As such, these results may not be able to be generalized to IL-6 inhibitor treatment approach.

## Conclusions

Our results coupled with other observational studies suggest that initial therapy with a biologic may reduce the use of glucocorticoids in hospitalized children with new-onset sJIA but is only utilized in a minority of patients. Further studies are needed to assess other important clinically relevant long-term outcomes that may be related to glucocorticoid use or avoidance in sJIA, including hospital readmissions for disease flares or infection, MAS, sJIA-related pulmonary disease, and long-term glucocorticoid toxicity. These studies will help to develop a more evidence-based approach to glucocorticoid usage in sJIA. Finally, with continued growing evidence of improvement in clinically important outcomes in new-onset sJIA with first-line biologic therapy, future work should focus on identifying and addressing barriers to widespread implementation of this treatment strategy across diverse care settings.

## Data Availability

The datasets used and/or analysed during the current study are available from the corresponding author on reasonable request.

## Abbreviations

sJIA: Systemic juvenile idiopathic arthritis
MAS: Macrophage activation syndrome
CARRA: Childhood arthritis and rheumatology research alliance
ACR: American college of rheumatology
DMARDs: Disease-modifying antirheumatic drugs
NSAIDs: Non-steroidal anti-inflammatory drugs
CBC: Complete blood count
IQR: Interquartile range
SD: Standard deviation
OR: Odds ratio
CTP: Consensus treatment plan
ICD: International classification of diseases
p-score: Propensity score
CI: Confidence interval
PHIS: Pediatric health information system
CHOP: Children’s hospital of philadelphia
ICU: Intensive care Unit
RCT: Randomized controlled trial
